# Addition of Atrial Myopathy to HATCH Score for Predicting New‐Onset Atrial Fibrillation After Ablation of Atrial Flutter

**DOI:** 10.1111/pace.70044

**Published:** 2025-09-11

**Authors:** Teerapat Nantsupawat, Yanhui Li, Stephanie Li, Neeraj Sathnur, Supavit Chesdachai, Selcuk Adabag, David G. Benditt, Venkatakrishna N. Tholakanahalli

**Affiliations:** ^1^ Department of Internal Medicine Cardiovascular Division Chiang Mai University Chiang Mai Thailand; ^2^ Department of Medicine Cardiovascular Division Minneapolis VA Medical Center Minneapolis Minnesota USA; ^3^ Department of Medicine University of Minnesota Minneapolis Minnesota USA; ^4^ Department of Medicine Cardiovascular Division University of Minnesota Minneapolis Minnesota USA; ^5^ Department of Medicine Division of Public Health Infectious Diseases and Occupational Medicine Mayo Clinic Rochester Minnesota USA

**Keywords:** atrial fibrillation, atrial myopathy, flutter ablation, HATCH

## Abstract

**Background:**

The available risk prediction models are inadequate to identify a true low‐risk patient for developing new‐onset atrial fibrillation (AF) after typical atrial flutter (AFL) ablation. We aimed to determine whether adding markers of atrial myopathy to HATCH score (hypertension, age ≥75 years, transient ischemic attack/stroke, chronic obstructive pulmonary disease, and heart failure) can improve prediction of new‐onset AF after ablation of typical AFL.

**Methods:**

The study included 208 consecutive patients who underwent successful ablation of typical AFL at Minneapolis VA Medical Center and University of Minnesota Medical Center. Patients with history of AF prior to ablation were excluded.

**Results:**

Among the 208 patients, 76 (36.5%) developed new‐onset AF post AFL ablation. Mean follow‐up duration was 62 ± 31.8 months. HATCH score was not associated with new‐onset AF. When adding atrial myopathy (presence of at least one of the following: PTFV_1_ > 5000 µV*ms, interatrial block determined by biphasic inferior p wave with duration >120 ms, left atrium (LA) diameter ≥44 mm, or LA index ≥3 cm/m^2^) to HATCH score (HATCH‐A_2_), the combination was independently associated with new‐onset AF. The AF incidence between HATCH‐A_2_ score of 0–1 and ≥2 were 6.7% and 40.5%, respectively (Odds ratio 9.04, 95% confidence interval: 2.05–39.81, *p* = 0.004). Particularly, when HATCH‐A_2_ score was 0, none of the patients developed AF.

**Conclusions:**

Adding atrial myopathy to HATCH score improved predictability and could be used to delineate a true low‐risk patient of new‐onset AF after typical AFL ablation.

**Abbreviations**
AF: atrial fibrillation
AFL: atrial flutter
CIED: cardiac implantable electronic device
CTI: cavotricuspid isthmus
ECG: electrocardiogram
EGM: electrogram
OAC: oral anticoagulation
PTFV_1_
: P‐wave terminal force
TIA: transient ischemic attack

## 1 Introduction

Isthmus‐dependent atrial flutter (AFL) is commonly treated by cavotricuspid isthmus (CTI) ablation with a high long‐term success rate of 92%–95% [1], and consequently patients with isolated AFL without a prior history of atrial fibrillation (AF), are usually considered cured after successful AFL ablation. Further, although there are not as yet formal practice guideline recommendations, in such cases many physicians do not feel the need to continue oral anticoagulation (OAC). However, recent reports have shown a 13.9%–26.2% incidence of apparently new‐onset AF post CTI ablation, and up to 45% in a more intensively monitored subgroup of patients (i.e., either by >7 days/year Holter monitoring or by implanted cardiac devices) [2]. These latter findings have tended to shift practice toward continuation of OAC after ablation of isolated AFL. Inevitably, however, continued anticoagulation exposes not only those patients deemed at high‐risk of later developing AF, but also those thought to be at low‐risk, of both the expense of medications and potential bleeding complications.

Multiple studies have attempted to define markers that predict high AF‐risk following isolated AFL ablation; typically, these markers have included structural/valvular heart disease [2], increased left atrial (LA) diameter [2, 3, 4], obstructive sleep apnea [4], interatrial block (i.e., prolonged P‐wave) [5], ejection fraction <50% [1], chronic obstructive pulmonary disease [3, 6], and hypertension [2]. Other AF‐risk factors that might additionally be used include: increasing age, diabetes mellitus, heart failure, and obesity.

No single factor or risk score has been able to identify true low‐risk patients that may not need long‐term OAC post AFL ablation. In this regard, the HATCH score [7] consists of hypertension (1 point), age ≥ 75 (1 point), history of TIA/stroke (2 points), chronic obstructive pulmonary disease (1 point), and heart failure (2 points). It has been used in an attempt to predict AF post AFL ablation. However, the results have been imperfect.

Given that certain readily accessible markers of ‘atrial myopathy’ including LA enlargement, presence of interatrial block, and left/right atrial remodeling are well established risk factors for developing AF, we hypothesized that incorporating these parameters into HATCH score will improve the ability to differentiate individuals at high AF risk after AFL ablation from a patient subset at low‐risk.

## 2 Materials and Methods

### 2.1 Patient Selection

This study retrospectively reviewed findings in all patients who had isthmus‐dependent atrial flutter and underwent successful CTI ablation between January 1, 2006 and December 31, 2014 from University of Minnesota Medical Center in Minneapolis, and the Minneapolis VA Medical Center. Patients with prior history of AF or follow‐up duration less than 1 year were excluded from analysis to avoid selection bias due to loss to follow‐up.

### 2.2 Definitions

AF was defined to be present if documented by one or more of the following: (1) 12‐lead electrocardiogram (ECG); (2) Rhythm strip lasting > 30 s from telemetry, Holter recording, or an implantable cardiac monitor (ICM), with irregular RR intervals unrelated to evident ventricular or atrial ectopy and no discernable P‐waves; or (3) Electrogram (EGM) from a cardiac implantable electronic device (CIED) showing atrial high rate episode ≥175 bpm lasting ≥5 min [8]. The AF diagnoses were independently adjudicated by two electrophysiologists who were blinded of the patients’ profile.

Atrial myopathy definition in our study was ‘Any complex of structural, architectural, contractile or electrophysiological changes affecting the atria with the potential to produce clinically‐relevant manifestations’ [9]. Atrial myopathy was considered present if any one of the following criteria was present: LA anteroposterior (AP) diameter ≥44 mm, LA diameter index ≥3 cm/m^2^ of body surface area, PTFV_1_ > 5000 µV*ms, or interatrial block P‐wave [9, 10].

**FIGURE 1:**
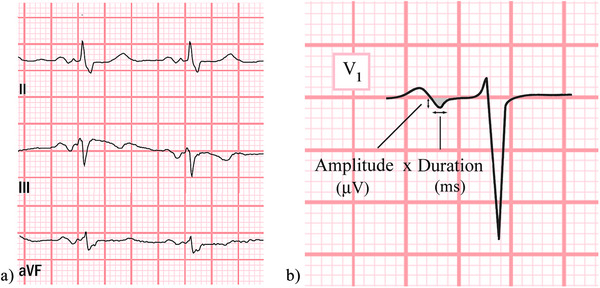
(a) The interatrial block: Typical ECG of interatrial block with P‐wave duration >120 ms and biphasic morphology in inferior leads [5]. (b) Illustration of ECG parameters used to calculate P‐wave terminal force (PTFV_1_), defined as the duration in milliseconds (ms) of the terminal part(negative) of the P wave in lead V_1_ multiplied by its depth in microvolts (µV*ms) [11]. One mm horizontally is equal to 40 ms, and 1 mm vertically is equal to 100 µV. [Colour figure can be viewed at wileyonlinelibrary.com]

Interatrial block was defined by P‐wave duration >120 ms and biphasic morphology in inferior leads (Figure 1a) [5]. P‐wave terminal force (PTFV_1_) was defined as the duration in milliseconds (ms) of the terminal part (negative) of the P wave in lead V_1_ multiplied by its depth in microvolts (Figure 1b) [11].

Echocardiographic analysis used a LA AP diameter cutoff of ≥44 mm for LA dilatation (from averaging LA AP diameter cutoff from multiple studies) [2, 3, 4, 12].

Routine follow‐up was defined as clinic visit in 1–2 months post AFL ablation procedure and then every 3–6 months with 12‐lead ECG at each visit. Holter/event monitor follow‐up comprised routine follow‐up plus at least one 24–48 h Holter monitor or 14–30 days event monitor. CIED follow‐up incorporated routine follow‐up plus regular clinic visit/remote monitoring of pacemaker, defibrillator, or implantable loop recorder every 6 months.

### 2.3 Predictor Variables

We collected baseline characteristics and pre‐specified risk factors for developing new‐onset AF, including age, LA AP diameter, obstructive sleep apnea, left ventricular ejection fraction, at least moderate severity valvular disease, chronic obstructive pulmonary disease, hypertension, CHA_2_DS_2_VASc, interatrial block p wave, PTFV_1_, atrial myopathy, HATCH and proposed HATCH‐A_2_ score. All predicting variables were collected at the time of the typical flutter ablation. ECGs for the criteria of atrial myopathy were assessed before the CTI ablation as the ablation can affect ECG especially P wave in inferior leads.

For HATCH score, points were assigned to each factor according to the original recommendation [13]. In evaluating the proposed HATCH‐A_2_ score, we conducted a multivariable logistic regression analysis to compare its predictive power against the HATCH score and atrial myopathy for the incidence of new‐onset AF following AFL ablation. Interactions among variables in the final model were assessed as well. We assigned 2 points to atrial myopathy and 1 point for each risk factor in HATCH because atrial myopathy demonstrated stronger association to subsequent new‐onset AF than 2 other risk factors combined.

### 2.4 Catheter Ablation

Isthmus‐dependent AFL was confirmed prior to ablation by 3D activation mapping using CARTO system (Biosense Webster). Entrainment was performed by pacing at coronary sinus proximal and distal electrodes, cavotricuspid isthmus site, and lateral right atrium. CTI ablation was performed using either 8 mm non‐irrigated (50–60 W, 60°C) or 3.5 mm irrigated (30–40 W) ablation catheter. Successful AFL ablation was confirmed by evidence of bidirectional block and differential pacing activation mapping.

### 2.5 Outcome Variables

Primary outcome was incidence of AF in the population post CTI ablation. Secondary outcome was incidence of ischemic stroke/systemic thromboembolism.

### 2.6 Statistical Analysis

Given the previously reported incidence of new‐onset AF post AFL ablation was about 25%, if the risk prediction model is to be able to divide patients into low‐risk group (risk of developing new‐onset AF of ≤5%) versus high‐risk group (risk of developing new‐onset AF of ≥30%), then 170 patients will provide statistical power of 95%. Chi‐square and *t*‐test were used to compare baseline characteristics. We used logistic regression to identify association/odds ratio of risk factors and new‐onset AF post AFL ablation. We performed multivariate logistic regression analysis by using pre‐specified risk factors for AF from literature reviews (obstructive sleep apnea, valvular disease, obesity, and diabetes mellitus) except factors that are parts of HATCH or HATCH‐atrial myopathy score (HATCH‐A_2_). All analyses were performed using SPSS Windows version 21, SPSS Inc. Chicago, IL USA.

## 3 Results

### 3.1 Baseline Characteristics

There were 231 consecutive patients with isolated typical AFL who underwent successful CTI ablation between January 1, 2006 and December 31, 2014. We excluded 23 patients who had follow‐up duration less than 1 year. Therefore, 208 patients were included in the analysis. Among the 23 patients with less than 1‐year follow‐up, there was only one patient who developed AF. Mean age was 64.8 ± 12.1 years old. Majority of patients were male (91.3%). Mean LA AP diameter and ejection fraction were 45.2 ± 7.2 mm and 49.2 ± 11.8%, respectively. Mean CHA_2_DS_2_VASc was 2.8 ± 1.5 with 79.3% of patients had score ≥2. There were 147 (70.7%) patients taking beta blockers and 14 (6.7%) patients prescribed antiarrhythmic drugs. There were no significant differences in baseline characteristics between patients who developed new‐onset AF and those who did not (Table 1). Mean follow‐up duration was 62.0 ± 31.8 months; the follow‐up duration ranged from 12 to 136 months. Of the 208 patients, types of follow‐up were routine office visit in 124 (59.6%) patients, CIED clinic follow‐up in 44 (21.2%) patients, Holter monitor in 24 (11.5%) patients, and event monitor in 16 (7.7%) patients.

**TABLE 1:** Baseline characteristics.

Baseline characteristics	New‐onset of AF		*p* value
	Yes (*N* = 76)	No (*N* = 132)	
Age (mean ± SD)	65.7 ± 10.4	64.3 ± 12.9	0.427
Male (%)	71(93.4)	119(90.2)	0.419
BMI (kg/m^2^) (mean ± SD)	31.8 ± 6.9	31.3 ± 6.7	0.634
OSA (%)	25(32.9)	42(31.8)	0.873
EF (mean ± SD)	48.3 ± 12.8	49.6 ± 11.1	0.437
CHF (%)	30(39.5)	40(30.3)	0.178
Valvular disease (%)	11(14.5)	20(15.2)	0.925
COPD (%)	18(23.7)	26(19.7)	0.498
HTN (%)	61(80.3)	100(75.8)	0.454
DM (%)	31(40.8)	50(37.9)	0.710
Prior TIA/stroke (%)	3(3.9)	6(4.5)	1.000
History CAD/PAD (%)	29(38.2)	55(41.7)	0.589
CHA_2_DS_2_VASc (mean ± SD)	2.9 ± 1.4	2.8 ± 1.6	0.657
0 (%)	3 (3.9)	8 (6.1)	0.104
1 (%)	10 (13.2)	22 (16.7)	
2 (%)	19 (25.0)	27 (20.5)	
3 (%)	15 (19.7)	38 (28.8)	
≥4 (%)	29 (38.2)	37 (28.0)	
Medications			
Beta blocker (%)	55(72.4)	92(69.7)	0.684
Antiarrhythmic (%)	7(9.2)	7(5.3)	0.279

Abbreviation: AP‐anteroposterior; BMI‐Body mass index; CAD‐coronary artery disease; CHF‐congestive heart failure; COPD‐chronic obstructive pulmonary disease; DM‐diabetes mellitus; EF‐ejection fraction; HTN‐hypertension; LA‐left atrium; OSA‐obstructive sleep apnea; PAD‐peripheral arterial disease; TIA‐transient ischemic attack.

### 3.2 Incidence of AF

Among the 208 patients in the study population, there were 76 (36.5%) individuals who developed new‐onset AF during follow‐up. Mean duration from CTI ablation to AF onset was 31.5 ± 26.3 months; the AF onset varied from 1 day to 8.9 years post‐ablation. New‐onset AF was detected earlier in Holter/event monitor and CIED group (22.0 ± 22.7 and 28.2 ± 22.6 months, respectively), compared to routine follow‐up group (37.4 ± 29.1 months), presumably due to the intensity of ECG recording in the former groups. Rate of AF detection was 38/124 (30.6%) in routine follow‐up, 13/40 (32.5%) in Holter/event monitor, and 25/44 (56.8%) in CIED follow‐up.

### 3.3 Risk Factors Predicting AF

As demonstrated in Table 1, a number of possible risk factors proved not to be associated with increased risk of new‐onset AF. Similarly, except for LA AP diameter, individual markers of atrial myopathy including interatrial block p wave, PTFV_1_, and LA diameter index had a trend toward increased risk of new‐onset AF from the univariate logistic regression analysis but not statistically significant (Table 2). However, when any one of these individual markers was used to define one entity, ‘atrial myopathy’, sensitivity to AF occurrences was increased and the combined marker became an independent predicting factor of new‐onset AF (OR of 3.92; 95% CI 1.84–8.38, *p* value < 0.001) (Tables 2 and 3). On the other hand, there was no cumulative effects from four different markers of atrial myopathy in predicting AF (data not shown).

**TABLE 2:** Univariate logistic regression analysis for risk factors predicting new‐onset AF post AFL ablation

Risk factors predicting AF	New‐onset of AF		OR (95% CI)	P value
	Yes (N = 76)	No (N = 132)		
LA AP diameter ≥44mm (%)	50/74 (67.6)	64/121 (52.9)	1.86 (1.02–3.39)	0.045*
LA diameter index (cm/m^2^) (mean ± SD)	2.1 ± 0.3	2.0 ± 0.4	1.23 (0.53–2.85)	0.625
Interatrial block (%)	14/71 (19.7)	13/126 (10.3)	2.14 (0.94–4.84)	0.070
PTFV_1_ >5000 uV^*^ms (%)	22/71 (31.0)	32/125 (25.6)	1.30 (0.68–2.48)	0.418
Atrial myopathy (%)	61/72 (84.7)	78/131 (59.5)	3.77 (1.82–7.82)	<0.001^**^
HATCH (mean ± SD)	2.1 ± 1.4	1.8 ± 1.3	1.18 (0.95–1.47)	0.128
CHADS_2_VASc (mean ± SD)	2.9 ± 1.4	2.8 ± 1.6	1.04 (0.87–1.25)	0.655
HATCH‐A_2_(%)			9.52 (2.20–41.23)	0.003*
0–1	2/72(2.8)	28/131(21.4)		
≥2	70/72(97.2)	103/131(78.6)		

Abbreviation: AF, atrial fibrillation; AFL, atrial flutter; AP, anteroposterior; HATCH‐A_2_, HATCH‐atrial myopathy; LA, left atrium; OR, odds ratio; PTFV_1_, p wave terminal force in V_1_.

* *p* value < 0.05

** *p* < 0.001.

**TABLE 3:** Multivariate logistic regression analysis for pre‐specified risk factors predicting new‐onset AF post AFL ablation.

Risk factors predicting AF	OR (95% CI)	*p* value
OSA	0.99(0.50–1.96)	0.970
Valvular disease	1.12(0.49–2.60)	0.783
Obesity (BMI ≥30 kg/m^2^)	1.51(0.76–3.02)	0.242
DM	0.83(0.43–1.60)	0.583
HATCH	1.13(0.90–1.42)	0.286
LA AP diameter ≥44 mm^a^	2.05(1.05–3.98)	0.035*
Atrial myopathy^b^	3.92(1.84–8.38)	<0.001^**^
HATCH‐A_2_ ≥ 2^c^	9.04(2.05–39.81)	0.004^*^

Abbreviations: AF, atrial fibrillation; AFL, atrial flutter; BMI, body mass index; DM, diabetes mellitus; HATCH‐A_2_, HATCH‐atrial myopathy; LA AP, left atrium anteroposterior; OR, odds ratio; OSA, obstructive sleep apnea.

* *p* value<0.05, ^**^ *p* < 0.001,

^a^ Model 1: OSA, valvular disease, obesity, DM, HATCH, LA AP diameter ≥44 mm.

^b^ Model 2: OSA, valvular disease, obesity, DM, HATCH, atrial myopathy.

^c^ Model 3: OSA, valvular disease, obesity, DM, HATCH‐A.

Neither CHA_2_DS_2_VASc score nor HATCH score was predictive of increased risk of new‐onset AF in both univariate and multivariate analysis. Two points were then assigned to the atrial myopathy factor in HATCH‐A_2_ score as described earlier in the method section. Adding atrial myopathy (2 points) to HATCH score (HATCH‐A_2_) with a cutoff value of 2, improved HATCH score predictability of new‐onset AF. When compared predictability of HATCH‐A_2_ score to atrial myopathy, HATCH‐A_2_ score with a cutoff value of 2 was overall better than atrial myopathy alone in identifying low‐risk patients (OR 9.04; 95% CI 2.05–39.81, *p* = 0.004 vs. OR 3.92; 95% CI 1.84–8.38, *p* < 0.001, and negative predictive value [NPV] of 93.3% vs. 82.8%, respectively) (Table 3).

**FIGURE 2:**
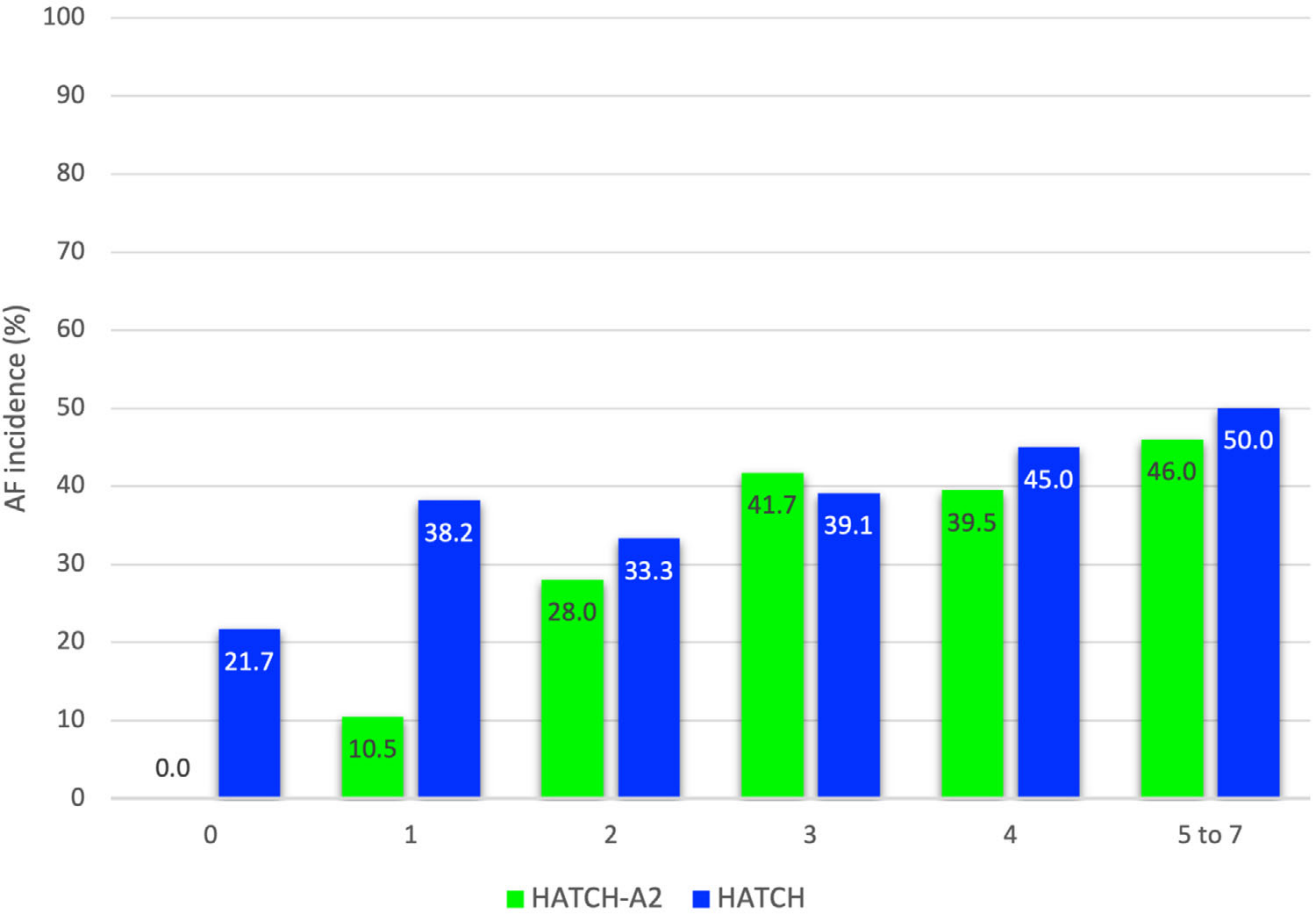
Bar chart demonstrating relationship between HATCH‐A_2_, HATCH score, and AF incidence. NS, non‐significance. [Colour figure can be viewed at wileyonlinelibrary.com]

**FIGURE 3:**
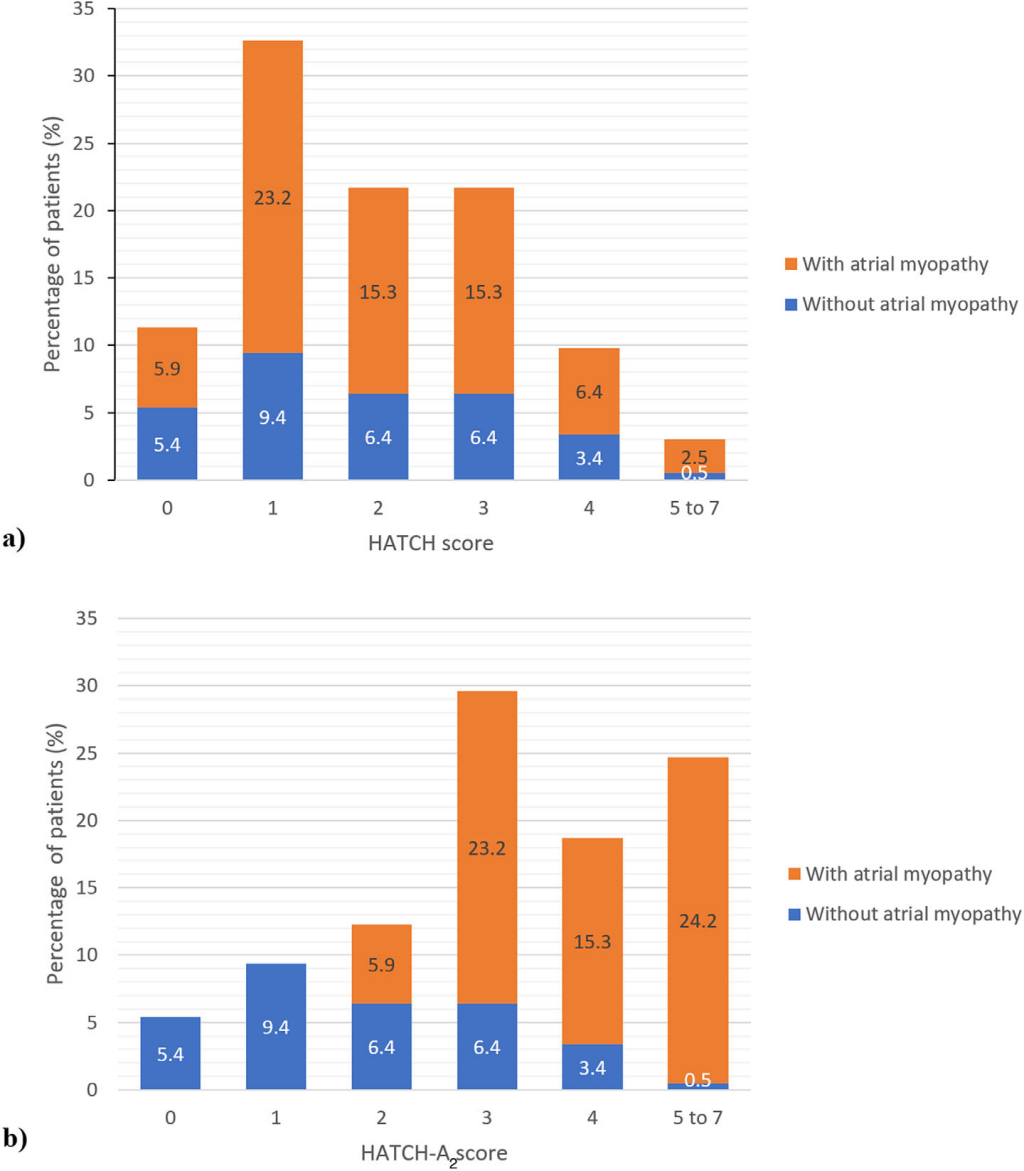
Proportion of atrial myopathy in each stratum of (a) HATCH score and (b) HATCH‐A_2_ score. [Colour figure can be viewed at wileyonlinelibrary.com]

When HATCH score was 0, there were five out of 23 patients (21.7%) who developed new‐onset AF as compared to 0 out of 11 patients (0%) when HATCH‐A_2_ score was 0 (Figure 2). AF incidence in patients with HATCH‐A_2_ 0–1 was 6.7% (2/30), as compared to 40.5% (70/173) in HATCH‐A_2_ ≥2 group. Although HATCH‐A_2_ score have improved discriminating ability as compared to HATCH score in low‐risk group, HATCH‐A_2_ score did not affect the risk stratification in the higher risk population when HATCH or HATCH‐A_2_ score were ≥2 (Figure 2). As demonstrated in Figure 3b, patients who had atrial myopathy would automatically had HATCH‐A_2_ score of 2 or more, and therefore left the HATCH‐A_2_ 0–1 group without atrial myopathy.

HATCH‐A_2_ cutoff value of 2 provided sensitivity and specificity in predicting new‐onset AF of 97.2% and 21.4%, respectively, with a negative predictive value of 93.3% and a positive predictive value of 34.5%.

### 3.4 Sensitivity Analysis

If we include 23 patients who had follow‐up duration of less than 1 year in the analysis. HATCH‐A_2_ ≥2 remained associated with a higher risk of new‐onset AF post AFL ablation (OR 8.66, 95% CI 2.01–37.32, *p* = 0.004).

### 3.5 Ischemic Stroke After CTI Ablation

Four patients (1.9%) or 3.72 per 1000 person‐years developed ischemic stroke with the onset of stroke from 0.2 to 40 months post ablation (Table 4). Two of them had new‐onset AF post AFL ablation but other two remained in sinus rhythm during follow‐up. All four patients had HATCH‐A_2_ score more than 2. Two patients had stopped OAC prior to ischemic stroke. Two patients developed ischemic stroke after ablation despite continuing OAC.

**TABLE 4:** Ischemic stroke patient's characteristics

ID	Develop AF	Device	Type of OAC	OAC stopped prior to stroke	CHA_2_DS_2_VASc	Prior TIA/stroke	HATCH‐A_2_ score	Time to stroke (mo)
1	No	Yes	None	Yes	1	No	4	8
2	Yes	Yes	warfarin	Yes	3	No	6	26
3	No	Yes	warfarin	No	3	No	3	0.2
4	Yes	No	warfarin	No	3	No	5	40

Abbreviations: AF, atrial fibrillation; HATCH‐A_2_, HATCH atrial myopathy; ID, identification; mo, month; OAC, oral anticoagulation; TIA, transient ischemic attack.

## 4 Discussion

### 4.1 Main Findings

This study provides four main findings regarding risk prediction of AF occurrence after successful AFL ablation. First, the incidence of new‐onset AF in patients post‐ablation of isolated AFL was 36.5% over 5 years which is similar to Chen et al. study with incidence of 39% [7]. Second, neither HATCH score nor CHA_2_DS_2_VASc score were able to predict new‐onset AF in patient after AFL ablation. Third, HATCH‐A_2_, a modified score system by incorporating the combined ‘atrial myopathy’ risk score to the HATCH score, greatly improved the sensitivity of predicting new‐onset AF post AFL ablation. Finally, in our study, mean duration post CTI ablation to AF onset was 31.5 months. This may represent actual delayed onset of AF, or delayed detection of AF, or both, but in either case emphasizes the importance of longer‐term follow‐up after apparently successful AFL ablation.

### 4.2 Relation of AF and AFL

When compared to AF incidence in a general population of 1.4%, the higher AF incidence in patients after ablation of typical AFL can be explained by the apparent close pathophysiologic interrelationship between AF and AFL. Typical AFL could be preceded by AF by either causing a functional line of block or initiating re‐entry around the preexistence anatomical line of block between the vena cava [14]. Atrial electrical and structural remodeling from atrial flutter, both of which could potentially be a substrate for AF, have also been shown in human studies [15].

### 4.3 HATCH and HATCH‐A_2_ Score

HATCH score was first developed as a tool to predict progression of paroxysmal AF to persistent AF [16]. Based on the multivariate analysis, clinical characteristics such as hypertension, age, previous transient ischemic attack or stroke, chronic obstructive pulmonary disease, and heart failure were shown to be independent factors in predicting AF progression and thus were included in the calculation of HATCH score. HATCH score was shown by Chen et al. to be a useful tool in predicting new‐onset AF following isolated AFL ablation [7]. However, in that study, patients with HATCH score of 0 and 1 still had AF incidence post ablation of 9.8% and 32.4%, respectively. In our study, 21.7% and 38.2% of patients with HATCH score of 0 and 1, respectively, had new‐onset AF post AFL ablation. There were significant portion of patients with HATCH score of 0 and 1 that had atrial myopathy (5.9% and 23.2%, respectively), which could explain why HATCH score failed to predict new‐onset AF post AFL ablation from our current study and the study from Garcia‐Seara et al. [13].

LA enlargement, presence of interatrial block, and left/right atrial remodeling are well established risk factors for developing recurrent AF post AF ablation, and accepted as markers of atrial myopathy. Our results showed that atrial myopathy was independently associated with new‐onset AF post CTI ablation. This is consistent with other atrial myopathy parameters studies [2, 3, 4, 5]. Although atrial myopathy alone was independently associated with increased AF incidence post AFL ablation, by adding atrial myopathy to HATCH score, we further improved the ability in identifying true low‐risk patients. Although with low specificity, HATCH‐A_2_ serves its purpose of providing a high sensitivity of 97.2% in predicting new‐onset AF as compared to HATCH score sensitivity of 59.2% when using a same cutoff value of ≥2.

### 4.4 Stroke

The incidence of ischemic stroke in our study was 1.9%, which is considerably lower than 5.85% reported by Exposito et al. on patients post AFL ablation [17]. Although mean CHADS_2_ score, post ablation OAC discontinuation rate, and AF incidence were similar, more patients in the study from Exposito et al. had prior stroke, which was the strongest risk factor for developing stroke in AF [18]. Despite of only four patients with ischemic stroke post AFL ablation, all of them had HATCH‐A_2_ score ≥2. The risk of developing new‐onset AF when HATCH‐A_2_<2 is 1.86 per 100 person‐years in our study, weighing against major bleeding from OAC of 3.57 per 100 person‐years [19]. Whether it is reasonable to consider discontinuing OAC post AFL ablation when HATCH‐A_2_<2, especially if patients have high bleeding risk, needs further validation in a large randomized control study.

### 4.5 Strengths and Limitations

The strengths of our study include relatively robust follow‐up methods post isolated AFL ablation incorporating routine clinic visits, Holter/event monitors, and when available CIED interrogation; these follow‐up techniques reflected a real‐world practice that has a mixture of clinical AF, subclinical AF, and also AF underdetection. A second strength was the relatively long mean follow‐up duration of 62 months, which increased the likelihood of detecting late‐onset AF post AFL ablation. Finally, the sample size and AF incidence were adequate to provide statistical power of >90%.

Several limitations of our analysis are important to consider. First, although the sample size had adequate statistical power, it was still relatively small skewed population with male predominance. Second, LA size was determined echographically by LA AP diameter in parasternal‐long axis, which is less accurate than LA volume. Third, post AFL ablation monitoring techniques were varied and lacked certainty regarding AF duration; the latter may have an impact on AF detection and stroke risk. Finally, about 60% of patients had routine clinic visit follow‐up and subclinical AF was possibly missed. Nonetheless, LOOP study demonstrated that detection of subclinical AF by implantable loop recorder and initiated OAC accordingly did not reduce the risk of stroke or systemic arterial embolism [20].

## 5 Conclusions

In our study, HATCH score did not predict new‐onset AF in patients after AFL ablation. Atrial myopathy is an independent risk factor for new‐onset AF post‐AFL ablation. When added atrial myopathy to HATCH score (HATCH‐A_2_), it further delineated true low‐risk patients in developing AF post‐AFL ablation.

## Author Contributions

T.N. designed the study, collected data, performed statistical analysis, interpreted the data, and wrote the manuscript. Y.H., N.S., S.C., S.L. collected and re‐checked the data prior to the analysis. J.L., V.T. designed the study, reviewed, and revised the manuscript. D.B., S.A. performed data interpretation and critically revised the manuscript. All authors agreed to be accountable for all aspects of the work in ensuring that questions related to the accuracy or integrity of any part of the work are appropriately investigated and resolved.

## Ethics Statement

The study protocol was approved by the Institutional Review Boards of all participating sites (STUDY00002032).

## Consent

The requirement for individual consent was waived.

## Conflicts of Interest

The authors declare that they have no pertinent commercial associations or sources of support that might pose a conflict of interest. Dr. Benditt was supported in part by a grant from the Dr. Earl E Bakken family in support of Heart‐Brain research.

## Data Availability

The data underlying this article are available in the article. The additional data will be shared on reasonable request to the corresponding author.

## References

[1] F. J. Perez , C. M. Schubert , B. Parvez , V. Pathak , K. A. Ellenbogen , and M. A. Wood , “Long‐Term Outcomes After Catheter Ablation of Cavo‐Tricuspid Isthmus Dependent Atrial Flutter: A Meta‐Analysis,” Circulation: Arrhythmia and Electrophysiology 2, no. 4 (2009): 393–401. 19808495 10.1161/CIRCEP.109.871665

[2] M. I. P. Waddah Maskoun , K. Ayoub , O L. Llanos , et al., “Incidence of Atrial Fibrillation After Atrial Flutter Ablation,” JACC: Clinical Electrophysiology 2, no. 6 (2016): 682–690. 29759746 10.1016/j.jacep.2016.03.014

[3] S. Raposeiras‐Roubin , J. Garcia‐Seara , P. Cabanas‐Grandio , et al., “Is Safe to Discontinue Anticoagulation After Successful Ablation of Atrial Flutter?,” International Journal of Cardiology 201 (2015): 631–632. 26343024 10.1016/j.ijcard.2014.12.154

[4] J. Voight , M. Akkaya , P. Somasundaram , et al., “Risk of New‐Onset Atrial Fibrillation and Stroke After Radiofrequency Ablation of Isolated, Typical Atrial Flutter,” Heart Rhythm 11, no. 11 (2014): 1884–1889. 24998999 10.1016/j.hrthm.2014.06.038

[5] A. Enriquez , A. Sarrias , R. Villuendas , et al., “New‐Onset Atrial Fibrillation After Cavotricuspid Isthmus Ablation: Identification of Advanced Interatrial Block Is Key,” Europace 17, no. 8 (2015): 1289–1293. 25672984 10.1093/europace/euu379

[6] J. G. Seara , S. R. Roubin , F. Gude Sampedro , et al., “Risk of Atrial Fibrillation, Stroke, and Death After Radiofrequency Catheter Ablation of Typical Atrial Flutter,” Clinical Research in Cardiology 103, no. 7 (2014): 543–552. 24566731 10.1007/s00392-014-0682-6

[7] K. Chen , R. Bai , W. Deng , et al., “HATCH Score in the Prediction of New‐Onset Atrial Fibrillation After Catheter Ablation of Typical Atrial Flutter,” Heart Rhythm 12, no. 7 (2015): 1483–1489. 25850017 10.1016/j.hrthm.2015.04.008

[8] G. Hindricks , T. Potpara , N. Dagres , et al., “2020 ESC Guidelines for the Diagnosis and Management of Atrial Fibrillation Developed in Collaboration With the European Association for Cardio‐Thoracic Surgery (EACTS): The Task Force for the Diagnosis and Management of Atrial Fibrillation of the European Society of Cardiology (ESC) Developed With the Special Contribution of the European Heart Rhythm Association (EHRA) of the ESC,” European Heart Journal 42, no. 5 (2021): 373–498. 32860505 10.1093/eurheartj/ehaa612

[9] A. Goette , J. M. Kalman , L. Aguinaga , et al., “EHRA/HRS/APHRS/SOLAECE Expert Consensus on Atrial Cardiomyopathies: Definition, Characterization, and Clinical Implication,” Europace 18, no. 10 (2016): 1455–1490. 27402624 10.1093/europace/euw161 PMC6392440

[10] H. Kamel , W. T. Longstreth, Jr , D. L. Tirschwell , et al., “The AtRial Cardiopathy and Antithrombotic Drugs in Prevention After Cryptogenic Stroke Randomized Trial: Rationale and Methods,” International Journal of Stroke 14, no. 2 (2019): 207–214. 30196789 10.1177/1747493018799981 PMC6645380

[11] H. Kamel , E. Z. Soliman , S. R. Heckbert , et al., “P‐Wave Morphology and the Risk of Incident Ischemic Stroke in the Multi‐Ethnic Study of Atherosclerosis,” Stroke; A Journal of Cerebral Circulation 45, no. 9 (2014): 2786–2788. 10.1161/STROKEAHA.114.006364 PMC4146624 25052322

[12] B. Frey , G. Kreiner , T. Binder , G. Heinz , H. Baumgartner , and H. D. Gossinger , “Relation Between Left Atrial Size and Secondary Atrial Arrhythmias After Successful Catheter Ablation of Common Atrial Flutter,” Pacing and Clinical Electrophysiology 20 (1997): 2936–2942. 12 Pt 1. 9455754 10.1111/j.1540-8159.1997.tb05463.x

[13] J. Garcia‐Seara , F. Gude Sampedro , J. L. Martinez Sande , et al., “Is HATCH Score a Reliable Predictor of Atrial Fibrillation After Cavotricuspid Isthmus Ablation for Typical Atrial Flutter?,” IJC Heart & Vasculature 12 (2016): 88–94. 28616550 10.1016/j.ijcha.2016.05.006 PMC5454134

[14] A. L. Waldo and G. K. Feld , “Inter‐Relationships of Atrial Fibrillation and Atrial Flutter Mechanisms and Clinical Implications,” Journal of the American College of Cardiology 51, no. 8 (2008): 779–786. 18294560 10.1016/j.jacc.2007.08.066

[15] S. Nattel , B. Burstein , and D. Dobrev , “Atrial Remodeling and Atrial Fibrillation: Mechanisms and Implications,” Circulation: Arrhythmia and Electrophysiology 1, no. 1 (2008): 62–73. 19808395 10.1161/CIRCEP.107.754564

[16] C. B. de Vos , R. Pisters , R. Nieuwlaat , et al., “Progression From Paroxysmal to Persistent Atrial Fibrillation Clinical Correlates and Prognosis,” Journal of the American College of Cardiology 55, no. 8 (2010): 725–731. 20170808 10.1016/j.jacc.2009.11.040

[17] V. R.‐E. F. Expósito , S. González‐Enríquez , G. Veiga , I. Olavarri , and J. J. Olalla , “Stroke and Systemic Embolism After Successful Ablation of Typical Atrial Flutter,” Clinical Cardiology 39, no. 6 (2016): 347–351. 27028600 10.1002/clc.22538 PMC6490731

[18] Stroke Risk in Atrial Fibrillation Working G. Independent Predictors of Stroke in Patients With Atrial Fibrillation: A Systematic Review. Neurology 2007;69(6):546–554. 17679673 10.1212/01.wnl.0000267275.68538.8d

[19] M. Lamberts , L. Staerk , J. B. Olesen , et al., “Major Bleeding Complications and Persistence With Oral Anticoagulation in Non‐Valvular Atrial Fibrillation: Contemporary Findings in Real‐Life Danish Patients,” Journal of the American Heart Association 6, no. 2 (2017): e004517. 28196815 10.1161/JAHA.116.004517 PMC5523754

[20] J. H. Svendsen , S. Z. Diederichsen , S. Hojberg , et al., “Implantable Loop Recorder Detection of Atrial Fibrillation to Prevent Stroke (The LOOP Study): A Randomised Controlled Trial,” Lancet 398, no. 10310 (2021): 1507–1516. 34469766 10.1016/S0140-6736(21)01698-6

